# Tinnitus in Children

**DOI:** 10.1007/s10162-024-00944-3

**Published:** 2024-05-06

**Authors:** Derek J. Hoare, Harriet Smith, Veronica Kennedy, Kathryn Fackrell

**Affiliations:** 1 NIHR Nottingham Biomedical Research Centre, Hearing Sciences, Mental Health and Clinical Neurosciences, School of Medicine, University of Nottingham, Nottingham, UK; 2 School of Clinical Therapies, College of Medicine and Health, University College Cork, Cork, T12 EK59 Republic of Ireland; 3 Department of Psychology, University of Sheffield, Sheffield, UK; 4 Bolton NHS Foundation Trust, Bolton, UK; 5 National Institute for Health and Care Research (NIHR) Coordinating Centre, School of Healthcare Enterprise and Innovation, University of Southampton, Southampton, UK

**Keywords:** Tinnitus, Child, Practice guidance, Research priority, Clinical questionnaires, Therapy

## Abstract

This perspective reviews the current state of the art and literature on tinnitus in children, prevalence and risk factors, clinical management, and future priorities for healthcare provision and research. Most research in the field to date appears to be prevalence studies, which have reached dramatically different estimates; this reflects the lack of a standard language when asking about the presence of tinnitus, or how bothersome, distressing, or negatively impacting it is for the child. Estimates are also likely affected by a lack of awareness of tinnitus amongst children and parents. Children are less likely to spontaneously report tinnitus than adults, and parents are often unaware their child could even develop tinnitus, considering it a disease of older age for example. It is critical that children are asked and learn about tinnitus. In hearing clinics, clinicians should routinely ask about all children about tinnitus and offer tinnitus care and settings that are child- and family-friendly. As well as asking directly, clinicians should be alert to soft signs of tinnitus such as unexplained listening, speech perception, concentration difficulties, worry or anxiety, or difficulties completing hearing tests or using hearing aids. The recently developed impact of Tinnitus in Children Questionnaire (iTICQ) can then be used to assess problems that are most commonly core to children’s experience of tinnitus. Clinical guidelines for tinnitus in children are few but provide recommendations for additional paediatric questionnaires and alternative assessments and for a range of treatment options. Of note, however, is the lack of clinical trials and, therefore, evidence of the effectiveness of any treatment for tinnitus in children. Significant and concerted work is therefore needed to raise awareness of tinnitus in children, understand the scale of clinical need, and standardise and evaluate clinical management options.

## Introduction

By consensus, *tinnitus* is defined as ‘the conscious awareness of a tonal or composite noise for which there is no identifiable corresponding external acoustic source’, which becomes *tinnitus disorder* ‘when associated with emotional distress, cognitive dysfunction, and/or autonomic arousal, leading to behavioural changes and functional disability’ [1]. That is, tinnitus is an auditory experience, whereas tinnitus disorder captures the auditory experience and the impact it has on a person’s quality of life. Children often describe tinnitus in similar terms to adults, i.e. ringing, buzzing, or chirping [2–4]. However, they can also use creative or emotive descriptors, such as sounds like ‘car beeping’, ‘rice crispies’, or ‘angry bees buzzing’, the latter perhaps more indicative of tinnitus that is distressing to the child [5]. Children and their families may also form narratives as they try to make sense of the tinnitus experience (e.g. tinnitus is caused by ‘a monster in their head’) [6]. However, several studies have shown that children do not often spontaneously report or discuss their tinnitus with adults but will present it when appropriately questioned [3–6]. Limitations in a child’s language and communication abilities present potential barriers to others becoming aware of the child’s tinnitus, recognising it as significant, and the child going on to receive care from health services. This highlights the need for greater awareness that tinnitus can affect children and the importance of health services tailored to the needs of children. 

This perspective reviews the current state of the art and literature on tinnitus in children, prevalence and risk factors, clinical management, and future priorities for healthcare provision and research.

## Prevalence, Risk Factors, and Pathophysiology of Tinnitus in Children

Tinnitus prevalence data suggest it may be as common in children as it is in adults and that some children experience tinnitus disorder (clinically significant tinnitus that negatively affects their day-to-day life) [3, 7]. A 2022 systematic review of tinnitus in children and adults by Jarach et al. [8] reported estimates of tinnitus prevalence in children ages 5–17 years ranging from 8.5 to 21% (pooled estimate of 13.0%) and a pooled estimate of ‘severe tinnitus’ in children of 2.7%. Interestingly these estimates were higher than those for young adults (aged 18–44 years, prevalence of 9.7% for ‘any tinnitus’ and 0.4% for ‘severe tinnitus’). The variability of estimates in children and these differences according to age is at least partially explained by the questions asked and definitions of tinnitus used; Jarach et al. concluded that data on tinnitus in children are more prone to different interpretations of the question used to assess tinnitus, that children are perhaps more frequently asked about tinnitus without specifically mentioning it by name, and that it is generally inadequately assessed in paediatric populations. In an earlier systematic review by Rosing et al. [9], children as young as 3 years old were reported to experience tinnitus. The prevalence estimates reported in that review ranged from 4.7 to 46%, higher (from 23.5 to 62.2%) in children with hearing loss, and the prevalence of ‘troublesome tinnitus’ in children varied from 0.6 to 49.2%. A subsequent study by Humphriss et al. [7], which aimed to determine the number of children with clinically significant tinnitus (defined as bothersome tinnitus that is more than seconds in duration), found a prevalence rate of 3.1%, so close to the lower estimates in earlier studies.

Rhee et al. [10] investigated the prevalence of tinnitus and associated risk factors in adolescents, in a nationwide sample of 1593 middle and high school students in South Korea. The prevalence of tinnitus was 46.0% and of severe tinnitus was 9.1%. Associated risk factors were older age, female gender, history of ear infection and sinusitis, leisure noise exposure, gaming, alcohol consumption, and cigarette smoking. Adolescents with tinnitus were also found to suffer more physical and mental health problems than those without tinnitus.

Further prevalence estimates were reported for Danish children aged 10 to 16 years by Nemholt et al. [11]. Their study involved 501 children recruited from eight mainstream schools. Using broad tinnitus questions, they estimated the prevalence of ‘any tinnitus’ to be 66.9%, the prevalence of ‘spontaneous tinnitus’ to be 53.7%, and of ‘noise-induced tinnitus’ to be 35.7%. Furthermore, they estimated that 34.6% and 2.4% of the children with ‘any tinnitus’ had ‘bothersome’ or ‘severely bothersome’ tinnitus. In terms of risk factors, females were nearly three times more likely than males to be bothered by tinnitus, and the odds of having hyperacusis were nearly five times higher in children who reported ‘spontaneous tinnitus’.

The largest prevalence study and arguably the most reliable estimates of tinnitus in a general child population came from Raj–Koziak et al. [12]. They performed audiometry and collected child and parent reports of tinnitus for 43,064 children aged 11 to 13 years old in Poland. They found that 3.1% of children reported tinnitus that was either constant or was experienced regularly and that constant or regular tinnitus was significantly more frequent (9%) in children who had hearing loss. A further 28% of children had experienced tinnitus briefly on occasion.

The prevalence of tinnitus in a clinical child population was estimated in a hospital-based study in Nigeria [13]. Of 2123 children seen in an ear, nose, and throat clinic, 132 (6.2%) presented with tinnitus. Likely causes of tinnitus were identified as febrile illnesses, otitis media, noise exposure, impacted earwax, ototoxicity, or of unknown cause. There was some evidence that prevalence in children increases with age, with the highest prevalence seen in 11–15-year-olds. In a further hospital-based study, the prevalence of tinnitus and hearing loss were determined in a caseload of children with COVID-19 infections [14]. Of the 192 children who recovered from COVID-19, 20 reported tinnitus, 16 reported hearing loss, and eight reported both hearing loss and tinnitus (prevalence was 14.6%). The authors concluded that neurological features like hearing loss and tinnitus can be found in children with COVID-19 infection and that more studies are required to confirm the underlying pathophysiology in children with COVID-19 infection, although their estimates fell well within the range seen in other child populations.

The most recent prevalence study identified the estimated prevalence of tinnitus and hyperacusis and associated hearing abilities and listening behaviours in children aged 9–12 years in Flanders [15]. The study involved a questionnaire distributed to 415 children in four different Flemish schools and determined the prevalence of permanent tinnitus in the sample to be 10.5%. Some children in this study also reported tinnitus causing anxiety (20.1%), problems with sleep (36.5%), and problems with concentration (24.8%). Notable also was that one-third of children reported high levels of daily noise exposure using personal listening devices, and less than half reported ever using hearing protection.

Variations in the prevalence estimates of tinnitus and tinnitus disorder in children likely reflect differences in study design, study populations, and particularly how tinnitus was defined and measured using various unvalidated approaches. There is a clear need for a standardised use of language and of tools that detect tinnitus and detect and measure tinnitus disorder. One further issue that will affect estimates is awareness and reliance on spontaneous reports by the child. A survey of parent knowledge conducted by the British Tinnitus Association (now Tinnitus UK) found that only 32% believed children under the age of 10 can have tinnitus, and only 37% believed it can affect children aged 10–16 years old (patient.info/news-and-features/signs-of-tinnitus-to-look-out-for-in-children). Reflecting this, Raj–Koziak et al. [12] found that, whereas 3.1% of children reported tinnitus, only 1.4% of parents reported that their child had spoken to them about having tinnitus. So, whilst we might work towards a consensus or evidence-driven method of assessing tinnitus and measuring the effects of tinnitus on the child, clinicians and researchers also need to be pro-active in asking the child about their experience of tinnitus.

In terms of pathophysiology, the precise mechanisms underlying subjective tinnitus in children are no more fully understood in children as they are in adults. Research suggests that irreversible cochlear damage, caused by excessive noise exposure, ageing, infection, head and neck trauma, and ototoxic drugs, is a likely trigger for tinnitus. Cochlear damage reduces input from the ear to the auditory brain, causing change in neural connectivity or activity (e.g. cortical reorganisation, increased central gain) which results in tinnitus [16]. This may explain the strong associations seen between hearing loss and tinnitus. Whilst pathophysiology has been theorised and studied relatively extensively in animal models [e.g. 17–19], translating into research involving adults who have tinnitus to either test hypotheses derived from animal models or to determine effects of intervention on putative tinnitus-related physiology [e.g. 20–22], such lines of study have yet to extend to tinnitus in children.

Hearing loss as a risk factor for tinnitus in children has been demonstrated in some studies, however. In a cross-sectional study on children aged 5–18 years who attended a tertiary care teaching hospital in Eastern India with a primary complaint of hearing impairment, Swain and Baliarsingh [2] examined the relationship between pure tone audiometry (up to 8 kHz) and tinnitus (assessed by questionnaire). Of the 172 children enrolled in the study, 104 self-reported tinnitus, of whom 67 (64.4%) were subsequently diagnosed with hearing loss. Of the 68 children who did not report tinnitus, just nine (13.2%) were diagnosed with hearing loss, indicating a strong association and likely causative factor.

A more extensive evaluation of causative or predictive factors for tinnitus was conducted by Levi et al. [23] in a retrospective chart review of 248 children and young people aged 1–19 years old, who presented with tinnitus in a tertiary paediatric hospital in the USA. Data included demographics, symptoms, historical data, imaging, and laboratory results, and these were compared with normative data. In contrast to some previous studies, their tinnitus sample had the same prevalence of dizziness as the general population, a lower incidence of otitis media, a lower prevalence of Eustachian tube dysfunction, otitis media, and headaches, but a *higher* incidence of rhinosinusitis. The authors identified the latter as a gap in the literature on tinnitus co-factors. They also suggested imaging children who present with tinnitus and hearing loss, and whether psychiatric diagnoses are associated with tinnitus in younger children as important further lines of research. Their sample had the same prevalence of hearing loss as the general child population. This is in line with some prevalence studies which find hearing loss is not more common in children who experience tinnitus than do not; although as discussed later, evidence is mixed [9].

Furthermore, a neuroimaging review concluded the aetiologies of continuous (as opposed to pulsatile) tinnitus in children are frequently associated with pathologies of middle and inner ear structures including vestibular schwannomas, cholesteatomas, trauma, Chiari malformations, and labyrinthitis ossificans [24, see also for a review of imaging for pulsatile tinnitus in children]. They propose computed tomography and magnetic resonance as complementary in paediatric tinnitus assessment, the former best to evaluate the integrity of the temporal bone structures and the latter to investigate the presence of masses or malformations and assess the vestibulocochlear nerve. Indications for imaging were not specified; although according to the British Society of Audiology (BSA) tinnitus in children guidance [5], these would include pulsatile tinnitus, unilateral tinnitus, or asymmetrical bone conduction thresholds, or suspicion of vestibular schwannomas or palatal myoclonus.

A final study to mention here is one conducted in Serbia examining the relationship between dietary factors and tinnitus in school-going adolescents aged 15–19 years [25]. From 1287 invitees, 1003 respondents completed a tinnitus screener questionnaire and food frequency questionnaire designed for the study. Logistic regression revealed the risk of tinnitus increased with increased intake of white bread, carbonated drinks, and foodstuffs classified as ‘fast food’. In contrast, there was a strong negative correlation between consumption of fresh vegetables and fruits and the presence of tinnitus, i.e. a ‘good’ diet (whether singularly or as part of some combination of factors) appears protective.

In summary, whilst tinnitus and tinnitus disorder appear to be common in children (comparable with rates in adults), estimates vary dramatically, in part due to differences in how it is diagnosed and the need to use age- and ability-appropriate language. Even then there is concern that tinnitus goes undiagnosed or a tinnitus-related issue goes misdiagnosed, e.g. as a problem with auditory processing. Potential risk factors identified in the literature include pathologies of middle and inner ear structures, bone or nerve malformations, rhinosinusitis, and social factors such as poor diet. However, whether these are mediating or moderating factors of tinnitus in children has yet to be modelled.

## Clinical Guidelines

By far, most tinnitus research to date has addressed how it manifests and how it might or can be managed in adults. This includes a vast and ever-increasing body of scientific evidence including systematic reviews of treatment efficacy (all in adults) [26–29], which has informed national and international clinical practice guidelines [30–32]. However, the research evidence base on tinnitus in children is small, largely composed of prevalence studies, as discussed earlier, with few studies addressing how tinnitus impacts the child or evaluating treatments (discussed in detail in later sections). This issue was highlighted in a 2013 UK-led tinnitus research prioritisation exercise where the question ‘What is the optimal set of guidelines for assessing children with tinnitus?’ was judged by tinnitus patients and clinicians to be one of the top 10 priority research questions on tinnitus [33]. However, the reality is that there were *no* national guidelines for the management of tinnitus in children at that time. This instigated production of the first national guidance on tinnitus in children by the BSA, which was published in 2015 [5]. Kentish and colleagues based this initial guidance on the limited scientific evidence available and the clinical experiences of the authors and other stakeholders. Recommendations for assessment and treatment are discussed later, but of note, the authors highlighted the lack of research and an urgent need for studies to determine the most effective approaches to assessing and treating tinnitus in children. The ethos of the BSA guidance is the need to take a child-friendly approach that puts the child at the heart of the management process and to provide care in settings that are tailored and appropriate to the needs of children and their families. It asks that clinicians listen respectfully to the child and communicates at the child’s developmental and linguistic level all the time with an awareness of what factors are likely to influence how the child communicates with them. It also describes using activities such as play and drawing to communicate complex ideas to the child.

More recently, the UK’s National Institute for Health and Care Excellence published its guidance on commissioning tinnitus services for both adults and children [32]. Guidance specific to children can be summarised briefly: provide tailored and age-appropriate reassurance and information and use age- or ability-appropriate measures (such as a visual analogue scale) for children and young people to assess how tinnitus affects them. The guidance strongly recommends that clinicians are alert at all stages of care to the behavioural and psychological wellbeing of all children and young people presenting with tinnitus, to talk to them and their family members or carers about how they feel, and to manage depression (or appropriately refer onwards for management) if identified [32].

Despite the publication of these two national guidelines for the management of tinnitus in children in the UK, to our knowledge, no other such national guidelines have been produced outside the UK, or at least that are findable in the public domain. There is a clear need to understand child services for tinnitus in different settings, countries, and cultures, and for appropriate guidance documents to be developed, not least to further inform and propagate research activity in this field.

## Investigation and Diagnosis

There is currently no objective way to detect or diagnose tinnitus. Psychoacoustic tests, such as loudness or pitch matching that aim to characterise the tinnitus sound, have demonstrated limited use due to poor reproducibility or simple correlation with subjective self-report measures of tinnitus loudness or distress [34, 35]. Clinically useful assessment of tinnitus therefore relies on self-report that the patient hears tinnitus, what tinnitus-related problems they are experiencing and how much these problems are impacting them. Whilst the tinnitus-related problems experienced by adults are well documented [36, 37], the breadth of problems and impact of tinnitus on children is much less so [38]. Although some tinnitus-related problems are likely common to both adults and children, the social context of the child is different, i.e. within their family, friendships, school, and community, and so the full range of problems, and their relative importance to the child will differ to that of adults, and indeed to children of different ages and stages of development. In their scoping review, Smith et al. [38] identified sleep difficulties, emotional difficulties, and concentration and hearing problems at school as being prominent. The review also concluded that problems relating to the impact of tinnitus on quality of life and feelings of isolation are important problem domains to consider when managing a child who has tinnitus. Swain and Baliarsingh [2] also suggest tinnitus can have a negative effect on social interactions and learning in children.

The review by Smith et al. [38] included 14 records reporting the use of structured questions to assess tinnitus problems in children and one record that used both clinician interviews and structured questions for this purpose. Where structured approaches were used, few records reported the actual question wording used. Two records reported the use of adult tinnitus questionnaires, the Tinnitus Handicap Inventory (THI) [39] and the Tinnitus Functional Index (TFI) [40], to assess the tinnitus in children, neither of which validate the use of these adult questionnaires for use with children. In a more recent scoping review, Tegg–Quinn et al. [41] identified six studies involving assessment of tinnitus in children using a patient-reported measure. Three of these were described as established measures, but none were developed specifically for children from which they concluded that, although clinicians are assessing children with tinnitus, this is being done without appropriate child-validated measures.

**Fig. 1:**
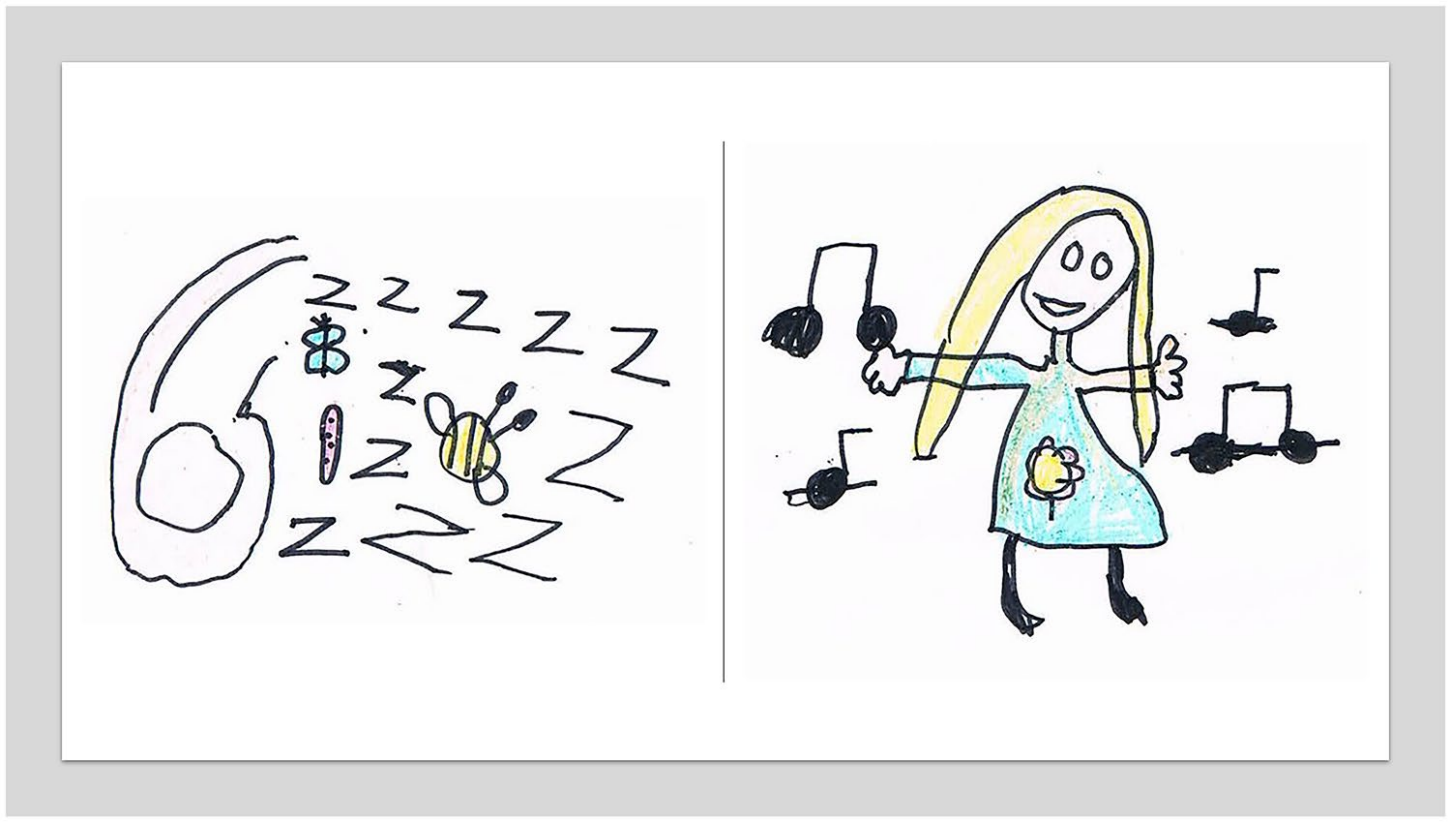
Images drawn by children in clinic to depict their experiences of tinnitus

So, what might optimal assessment involve? In addition to audiological assessment, assessment of tinnitus should involve thorough history taking and the use of appropriate assessment tools including questionnaire that are age- and ability-appropriate. The BSA tinnitus in children guidance [5] describes various strategies to ensure assessment of the child is conducted sensitively and optimally, according to their specific cognitive and linguistic abilities, using non-judgmental questions to determine the impact of tinnitus on all aspects of the child’s life. In the absence of a child-specific tinnitus questionnaire at the time, the guidance recommends using validated paediatric questionnaires to screen for problems that may be related to tinnitus impacting psychological wellbeing or educational performance [5]. The Children’s Auditory Performance Questionnaire [42], the Listening Inventory For Education [43], the Paediatric Index of Emotional Distress Questionnaire [44], the Revised Children’s Anxiety and Depression Scale [45], the Strengths and Difficulties Questionnaire [46], and the Screening Instrument For Targeting Educational Risk [47] are all recommended. The BSA Guidance advised that items in adult tinnitus questionnaires could be used to facilitate discussion with older children but not to measure tinnitus distress. This is appropriate given adult questionnaire will for many children be overly complex and burdensome in terms of time to complete, reading level, and understanding response scales [48]. Single-item measures were also recommended to help children describe their level of tinnitus distress, in the absence of a suitable alternative multi-item scale. With young children, clinicians are encouraged to use techniques such as play and drawing to gain further information about the child’s tinnitus symptoms. Figure 1 shows some drawings created in clinic by children, used as part of their assessment discussions. Tools such as the Ida ‘My World’, a counselling tool designed to help children talk about their experience of hearing loss (idainstitute.com/tools/my_world), can equally be useful for assessing tinnitus [49]. It provides a board-game style platform for children to show you how they go through their day and where they have their greatest successes and difficulties.

We recently published the first self-report questionnaire measure of the impact of Tinnitus in Children Questionnaire (iTICQ) [50]. It was developed through a rigorous iterative process of determining which problems were core to children’s experience of tinnitus, item development, readability assessment, cognitive interviews, and initial psychometric validation. It was designed and tested to be completed independently by children aged 8 to 16 years old. The iTICQ contains three scene-setting items which are not scored, and 33 items divided across six problem subscales: *sleep and feeling tired*, *learning*, *emotional health*, *hearing and listening*, *taking part*, *and relationships*. For each item, the child is asked to select the option that is most relevant to them based on how frequently they experienced X (e.g. felt annoyed because of tinnitus, felt tired because of tinnitus) over the last 2 weeks. Response options are on a 5-point scale from ‘none of the time’ to ‘all of the time’, with reverse marking of positive valence items such that higher overall scores indicate a greater impact of tinnitus. Initial evaluation of the iTICQ shows it has good content validity and can be used to measure the impact of tinnitus across problem domains that are most relevant to children with tinnitus [49].

## Treatment

Many of the tinnitus treatments recommended for children are the same as those used for adults although the evidence base is much smaller [5, 32]. For example, cognitive behavioural therapy for tinnitus is recommended for adults and children, and whilst it has been the subject of approximately 30 randomised controlled trials in adults, it has yet to be trialled in children [26]. Treatments recommended for children include advice and information giving, hearing aids, wearable sound generators, non-wearable sound enrichment, sleep hygiene, relaxation techniques, mindfulness techniques, and cognitive behavioural therapy [5]. Narrative therapy for tinnitus is uniquely recommended for children. With this form of psychotherapy, the child can develop narratives or stories about themselves that help make sense of their lives and what happens to them [51]. A key component of narrative therapy is the externalisation of problems, putting them ‘outside the person’, e.g. by drawing them or giving them (the problems) names. This allows for the exploration of other stories that challenge or contradict the ‘problem’, e.g. a time when the child got to sleep well, even though they had tinnitus, or the creation of new stories and new ways of thinking about the problems, creating a new tinnitus narrative.

All these recommended treatments have found their way into some paediatric tinnitus clinics; a service evaluation in the UK found that 28% of services use cognitive behavioural therapy and/or mindfulness, and 15% use narrative therapy [52]. This means we could at a reasonable scale evaluate their real-world effectiveness. However, treatments are likely unstandardised, and there has been little research to evaluate their effectiveness in children.

A 2021 systematic review of tinnitus treatments in children by Dullaart et al. [53] found just one case report and five observational studies that met their eligibility criteria. Three studies applied counselling and sound therapy and reported improvement in tinnitus outcome (single question, clinical interview, or THI) in 68 out of 82 children after 3–6 months of treatment. Two studies reported drug treatments (betahistine, xylocaine) and reported improvement in self-devised single-item measures in 74 out of 86 patients after 10 days to 3 months of treatment. One study reported the outcome of biofeedback therapy, describing an improvement in tinnitus loudness and annoyance (assessed through clinical interview) after 2 months of treatment. The authors judged there to be a high risk of bias in all the included studies and thus the effectiveness of tinnitus treatments cannot be concluded. The authors called for randomised controlled trials with long-term follow-up.

A more recent systematic review by Deutsch [54] further evaluated the literature on evidence-based practice in the management of tinnitus in children and adolescents. The most common treatment methods were the use of hearing aids, sound therapy with sound generators, counselling, and education. Other methods discussed included referral to a mental health professional, tinnitus retraining therapy, addressing sleep disturbances, and relaxation strategies. Of the ten articles included in the review, only four reported the use of outcome measures to evaluate treatment effects, either questionnaire, interview, or parent survey, none of which were validated. Again, the effectiveness of treatments could not be concluded, and the author called for the creation and use of validated measures for future clinical trials. Similarly, in their scoping review, Tegg–Quinn et al. [41] concluded little evidence exists to support any clinical interventions.

Multiple studies have found that few children with tinnitus access specialist care [55–58]. Of concern, these studies also reported some clinicians were reluctant to discuss tinnitus with children because they feared causing unnecessary distress. Szibor et al. [58] reported an average 12-month delay between children in Finland first reporting tinnitus symptoms and their access to a specialist tinnitus clinic. Of those children who do receive treatment for tinnitus, many are managed in adult tinnitus services [55, 56, 59]. The need for more child-specific tinnitus services is therefore clear. Differences in children’s physical, emotional, and intellectual development have implications for their health care needs, both in terms of the treatment they receive or the setting in which they are cared for. When caring for children, the wider social context needs at school and other support systems need to be considered [60]. Smith et al. [52] identified that specialist staff training, access to child-specific tools, and the treatment and referral of children with tinnitus-related psychological problems represent key areas in need of optimisation.

## Conclusions and Research Priorities

Children are much less likely to spontaneously tell others about their tinnitus than adults, and when they do, they may struggle to articulate or use unfamiliar terms. A lack of awareness, whether among the child’s family, social, educational, or clinical environments, may worsen their tinnitus-related problems and impede their ability to access appropriate care. If a child reports their tinnitus to an adult who dismisses it or does not recognise its significance, the child’s worries or fears about tinnitus may increase. They may then be reluctant to report their tinnitus-related problems, deepening the lack of understanding of the child’s experience and behaviours. Clinicians must therefore be alert to ‘soft’ signs that a child has tinnitus. Examples may include parent reports of sleep difficulty, a child who is distressed or tries to avoid certain sound (quiet, noisy) environments, unexplained listening or concentration difficulties, anxiety or worry, and in hearing clinics it should be routine to ask children ‘if they hear noises in their ears or head’ [5]. In a concept mapping study by Tegg–Quinn et al. [61], both adults who experienced tinnitus as children and clinicians identified that recognising the occurrence of tinnitus for children and adolescents, acknowledging the potential for distress, and initiating clinical care are all key to effective management. Addressing the concerns and needs of parents was also considered important, and so the authors recommend approaching the management of tinnitus in children from a family-centred care framework. Within this, parents and children should also be asked about their worries and concerns individually as these may not be the same. The need for more general and specialist training in paediatric tinnitus management and the production, provision, and validation of child and family-friendly resources are indicated to increase awareness as well as provision of appropriate care. A starting point is the BSA tinnitus in children guidance [5] which provides various practical assessment and treatment options that can be selected to inform protocols of care for different groups of children (e.g. older children who can self-complete questionnaires, younger children who may need alternative ways of expressing tinnitus impact) situations (e.g. information for management of tinnitus in the classroom), and critically, guidance on development of a paediatric tinnitus service. Increased awareness of the scale of tinnitus problem in children will be needed among policy makers and health commissioners, to ensure child- and family-friendly services are supported and adequately resources.

Whilst many studies of the prevalence of tinnitus in children have been reported, these do not provide for a conclusive estimate of the scale of the problem or public health and healthcare needs. A major hurdle is the lack of a standardised, validated diagnostic criterion for tinnitus, and a validated measure of tinnitus distress. For the former, there is a need for coordinated efforts to produce by consensus standardised questions or items to constitute a child having tinnitus; these will likely differ for different ages and cultures. A precedent for this approach is reported by Biswas et al. [62] who did so to determine the prevalence of tinnitus in adults across 12 diverse European countries using a standardised set of tinnitus-related questions in country-specific languages. The latter issue, measuring tinnitus distress, could be fulfilled by the iTICQ, at least for 8–16-year-olds. Our preliminary validation suggests validity, reliability, and responsiveness of the questionnaire, but powered studies involving hundreds of children with tinnitus are required to formally establish its psychometric properties and thus its utility as a research tool, and its clinical and cost-effectiveness as a questionnaire as recommended by NICE [32]. Translation to languages other than English and validation in different populations will also be needed, as may cultural adaptations. Thereafter (or with appropriate revision), the iTICQ may have utility as a diagnostic tool and outcome measure, for use in clinic (for audit, treatment evaluation) and in clinical trials; a multi-item questionnaire is essential to the sensitive evaluation of novel interventions.

In terms of treatment research, *all* treatments or potential treatments for tinnitus in children require a series of development, feasibility, and efficacy/effectiveness studies. One of the key recommendations by NICE is the evaluation of the clinical and cost-effectiveness of psychological therapies for children and young people who have tinnitus-related distress [32]. Other research recommendations include determining whether relaxation strategies are clinically and cost-effective for the management of tinnitus for children and young people. These treatments are already in use in clinical practice, but a standardised protocol will need to be selected or developed as a starting point for evaluation through research.

Finally, there is evidence in the literature that indicates the need for concerted efforts to educate children to prevent tinnitus or exacerbation of tinnitus. Studies reviewed here highlight unsafe listening behaviours and a disregard or lack of understanding of hearing protection in children. Campaigns for safe listening and hearing protection are warranted.

## Data Availability

We do not analyse or generate any datasets in this review.

## Declarations

### Disclaimer

The views expressed are those of the authors and not necessarily those of the NIHR, the NHS, or the Department of Health and Social Care.
